# Targeted Brain Tumor Therapy by Inhibiting the MDM2 Oncogene: In Vitro and In Vivo Antitumor Activity and Mechanism of Action

**DOI:** 10.3390/cells9071592

**Published:** 2020-07-01

**Authors:** Surendra R. Punganuru, Viswanath Arutla, Wei Zhao, Mehrdad Rajaei, Hemantkumar Deokar, Ruiwen Zhang, John K. Buolamwini, Kalkunte S. Srivenugopal, Wei Wang

**Affiliations:** 1 Department of Pharmaceutical Sciences, Jerry H. Hodge School of Pharmacy, Texas Tech University Health Sciences Center, Amarillo, TX 79106, USA; Surendra.R.punganuru@ttuhsc.edu (S.R.P.); Viswanath.arutla@ttuhsc.edu (V.A.); 2 Department of Pharmacological and Pharmaceutical Sciences, College of Pharmacy, University of Houston, Houston, TX 77204, USA; wzhou20@Central.UH.EDU (W.Z.); mrajaei@Central.UH.EDU (M.R.); rzhang27@central.uh.edu (R.Z.); 3 Department of Pharmaceutical Sciences, College of Pharmacy, Rosalind Franklin University of Medicine and Science, North Chicago, IL 60064, USA; h.deokar@rosalindfranklin.edu (H.D.); john.buolamwini@rosalindfranklin.edu (J.K.B.); 4 Drug Discovery Institute, University of Houston, Houston, TX 77204, USA

**Keywords:** MDM2 inhibitors, p53-independent, temozolomide, combination therapy, brain tumor

## Abstract

There is a desperate need for novel and efficacious chemotherapeutic strategies for human brain cancers. There are abundant molecular alterations along the p53 and MDM2 pathways in human glioma, which play critical roles in drug resistance. The present study was designed to evaluate the in vitro and in vivo antitumor activity of a novel brain-penetrating small molecule MDM2 degrader, termed SP-141. In a panel of nine human glioblastoma and medulloblastoma cell lines, SP-141, as a single agent, potently killed the brain tumor-derived cell lines with IC_50_ values ranging from 35.8 to 688.8 nM. Treatment with SP-141 resulted in diminished MDM2 and increased p53 and p21^cip1^ levels, G2/M cell cycle arrest, and marked apoptosis. In intracranial xenograft models of U87MG glioblastoma (wt p53) and DAOY medulloblastoma (mutant p53) expressing luciferase, treatment with SP-141 caused a significant 4- to 9-fold decrease in tumor growth in the absence of discernible toxicity. Further, combination treatment with a low dose of SP-141 (IC_20_) and temozolomide, a standard anti-glioma drug, led to synergistic cell killing (1.3- to 31-fold) in glioma cell lines, suggesting a novel means for overcoming temozolomide resistance. Considering that SP-141 can be taken up by the brain without the need for any special delivery, our results suggest that SP-141 should be further explored for the treatment of tumors of the central nervous system, regardless of the p53 status of the tumor.

## 1. Introduction

Cancers of the central nervous system (CNS) include both the primary brain cancers that occur in children and adults (about 27,000 cases diagnosed in the USA per year) and the brain metastases originating from other malignancies (>120,000 cases/year) [1,2,3,4]. Among the pediatric cancers, brain tumors rank second in frequency and cause of mortality, following leukemias [5]. Glioblastoma multiforme (GBM) is the most common and lethal primary brain cancer in children and adults, and the therapeutic outcome for these highly invasive and aggressive tumors continues to be dismal [6]. The survival of GBM patients is only about 3 months without any treatment, however, even with aggressive surgery, radiation, and chemotherapy, the median survival does not typically reach beyond 12–15 months [1,6]. Patients with childhood medulloblastoma fare better, but with a high cost to their quality of life [5]. There are concerns about using radiation for brain cancers in younger patients because of the development of long-term effects, such as neurocognitive deficits, endocrinopathy, and cerebral vasculopathy [5]. In these cases, chemotherapy is the sole and palliative choice. Chemotherapy using small molecular weight hydrophobic alkylating agents that cross the blood–brain barrier is a mainstay in the therapeutic management of brain cancers [7]. Unfortunately, drug resistance is a major hurdle to brain tumor therapy using alkylating agents. Understanding and targeting the molecular mechanisms responsible for chemoresistance is necessary to improve the treatment outcomes. Inhibiting pathways associated with drug resistance should improve the therapeutic response, and thus extend patient survival. Developing an effective and safe treatment for brain cancer, and determining ways to reduce drug resistance, remain unmet medical needs.

As with the majority of human cancers, a disruption of the p53 pathway leading to the emergence of oncogenic genomes is a common feature of glioblastomas and lower-grade gliomas [8,9,10,11,12,13,14]. In fact, multiple points within the p53 signaling pathways are disrupted in GBM due to missense mutations and/or amplification, the overexpression of MDM2, and/or loss of expression of the p16Ink4-p14ARF locus, all of which block p53 activity and lead to uncontrolled glial cell proliferation and brain oncogenesis. The most common *p53* mutations found in GBM are point mutations that target the conserved domains of *p53* in exons 5, 7, and 8, which are crucial for its DNA binding. These mutations lead to high-level expression of gain-of-function variants of the tumor suppressor [8,13]. Under normal conditions, the MDM2 feedback loop precisely regulates the level of p53 activity and the duration of p53 activation in response to DNA damage and various metabolic/pathological stresses by targeting p53 for degradation through its intrinsic E3 ubiquitin ligase activity [15,16]. In addition, activation of p53 can be achieved through the inactivation of MDM2 by p14 ARF binding [13]. MDM4 or MDMX, a member of the MDM2 family, can also regulate the activity of p53 either by itself or via heterodimerization with MDM2 [13].

Deregulation of the *p53* tumor suppressor occurs in >85% of primary glioblastomas in the form of *p53* gene mutations (35%) or homozygous deletion of the *p16Ink4*/*ARF* locus (60%), while amplification of the *MDM* homologs 1/2/4 has been observed in 14% of patients with this cancer type [8,9,10,11,12,13]. Most primary glioblastomas and recurrent glioblastomas possess at least one defect along the p53- p16Ink4A-p15Ink4b-p14ARF-MDM2 axis and these abnormalities have been implicated in the proliferation, invasion, migration, apoptotic escape, stem cell properties, drug resistance, and response to therapies of GBM [10,13]. MDM2 and MDM4 also appear to have important roles in normal CNS development as well, because conditional knockout mouse models showed that a loss of MDM2 in the growing brain results in massive p53-dependent apoptosis and degeneration of the neuroepithelium, hydrocephalus, and perinatal lethality [12,15]. Recent findings showed that p53 also controls the proliferation, differentiation, and survival of stem cells, highlighting the relevance of p53 in the pathophysiology of GBM [15].

Glioblastoma and other brain tumors are heterogeneous neoplasms with multiple genetic abnormalities that typically require several therapeutic “hits” to achieve effective elimination. MDM2, as an important hub for cell survival, growth, invasion, and DNA repair [16,17], remains a major therapeutic target in this tumor type. The Cancer Genome Atlas (TCGA) estimates that amplification of *MDM2* and *MDM4* that effectively suppresses the biological functions of p53 are found in 14% and 7% of GBMs, respectively [18,19], and such overexpression only occurs in cells harboring the wild-type p53 protein. Consequently, MDM2 inhibition has emerged as a prime therapeutic strategy to reactivate the p53 pathway. This reactivation leads to cell cycle arrest, increased apoptosis, and decreased tumor growth. In this context, pharmacological interventions to suppress MDM2 expression, inhibit the p53–MDM2 interaction, and curtail the E3 ubiquitin-ligase activity of MDM2 have been investigated. Nutlins were the first such molecules identified through a chemical library screen [20], and the analog RG7112 was subsequently characterized as an MDM2 inhibitor [21]. Several other MDM2 inhibitors, such as RG7388, MI77301, CGM097, MK8242, and AMG232, have been investigated for therapeutic effects against human cancers, with a few of them having been evaluated in CNS malignancies [22,23,24,25,26,27,28]. Some studies have suggested that MDM2 inhibition is a promising therapeutic strategy for treating GBM with wild-type p53 [22,23,24,25,26,27]. Considering that the majority of GBM harbors mutant p53, small molecule MDM2 antagonists are expected to have low or no efficacy against these types of GBM. In addition, the low permeability of the blood–brain barrier and the poor distribution into the brain have limited the efficacy of some MDM2 inhibitors against GBM [28]. Therefore, there is a need to identify a novel, p53-independent strategy to inhibit MDM2 that can effectively reach sufficient concentrations in the brain. We recently reported the discovery, characterization, and anticancer efficacy of a unique new class of MDM2 inhibitor, SP-141. SP-141 is a first-in-class MDM2 inhibitor with unique mechanisms of action different from the existing reported p53-dependent MDM2 inhibitors that have been evaluated in preclinical and clinical investigations. Specifically, SP-141 directly binds to the MDM2 protein, inhibits MDM2 expression, and induces its autoubiquitination and proteasomal degradation [29,30,31,32]. Because SP-141 can cross the blood–brain barrier fairly well [29] and can inhibit MDM2 in the presence or absence of wild-type p53 [29,31,32], the current study investigated the effects of SP-141 in brain tumor cell cultures and orthotopic xenograft models of medulloblastoma and glioblastoma.

## 2. Materials and Methods

### 2.1. Cell Lines and Reagents

Human glioblastoma cell lines, U87MG, SNB19, U251, LN229, T98G (American Type Culture Collection; ATCC), GBM10 (Dr. Jann Sarkaria, Mayo Clinic), SF188 (Neurosurgery, University of California, San Francisco), UW18, UW28 (Dr. Francis Ali-Osman, Duke University), and LN229 (Dr. Erwin Van Meir, Emory University), and the medulloblastoma cell lines DAOY (ATCC) and UW228 (Dr. Ali-Osman, Duke University) were used for the present studies. Cells were maintained in Dulbecco’s Minimal Essential Media supplemented with 10% fetal bovine serum and antibiotics in a humidified atmosphere of 5% CO_2_ at 37 °C. SP-141 was synthesized as described earlier [30]. Temozolomide (TMZ), other chemicals, and biochemicals were of the highest grade available and obtained from Sigma-Aldrich Company.

### 2.2. In Vitro Assays for Cytotoxicity, Cell Migration, and Invasion

The cytotoxicity of SP-141 alone or in combination with TMZ was evaluated in glioma cells following a continuous 72 h drug exposure. The resazurin reduction assay was used as described previously [33]. Wound healing assays to evaluate cell migration were performed by growing tumor cells in 6-well plates. Scratches were made at experimental time zero and then the cells were exposed to varying concentrations of SP-141. The wells were photographed at different time points and the migration was quantified [30,31,32]. Transwell invasion assays were performed by exposing tumor cells to SP-141 for 24 h, and the cells that migrated into the lower chambers were stained with crystal violet [30,31,32].

### 2.3. Flow Cytometric Assay for MDM2 Degradation and p53 Activation

Tumor cells were treated with SP-141, trypsinized, and fixed with 0.5% glutaraldehyde as described previously [34]. Following NaBH_4_ reduction, cells were blocked, treated with corresponding antibodies in the presence of 0.2% Triton X-100, then incubated with FITC-conjugated secondary antibodies. The washed cells were analyzed by flow cytometry [30,31,32,34].

### 2.4. Cell Cycle Analysis and Detection of Apoptosis

Flow cytometry using propidium iodide (PI) was employed to assess the cell cycle progression as described in previous reports [30,31,32,33,34]. Annexin-V staining was performed after the drug-treated cells had been trypsinized and washed with PBS. The cells were resuspended in 1 mL of binding buffer containing 10 mM HEPES (pH 7.4), 2.5 mM calcium chloride, 1 mM magnesium chloride, and 4% bovine serum albumin. Aliquots of cells (500 µL) were mixed with 5 µL Annexin V-FITC (BD Bioscience) at room temperature for 30 min. The cells were then fractionated on a BD FACSCanto^TM^ II flow cytometer and cells positive or negative for Annexin-V and PI were quantitated [30,31,32,34].

### 2.5. Western Blotting

For direct Western blotting, cell pellets were trypsinized, washed with PBS, and sonicated in a Tris–HCl buffer (50 mM, pH 8.0) containing 4 mM 2-mercaptoethanol, 1% glycerol, 1 mM EDTA and protease inhibitors, 0.5 mM PMSF, and 2 mM benzamidine. After centrifugation, equal amounts of protein were run on 12% SDS–polyacrylamide gels and subsequently electro-transferred to Immobilon-P membranes. These blots were blocked with 5% non-fat dry milk dissolved in Tris-buffered saline for 2 h, followed by exposure to the respective primary and secondary antibodies. Enhanced chemiluminescence (ECL) was performed for band detection and band intensities were quantitated using the ImageJ software [30,31,32,34].

### 2.6. Animals and Development of Intracranial Glioblastoma/Medulloblastoma Xenografts

Female 4-week-old triple immune-deficient NCG (NOD CRISPR Prkdc IL2 receptor gamma) mice were purchased from Charles River Laboratories. The study was performed according to the guidelines of the Institutional Animal Care and Use Committee (IACUC), under Texas Tech University Health Sciences Center Protocol No. 07050 approved on 31 July, 2019 (Animal Welfare Assurance Number A3056-1). Human medulloblastoma DAOY cells and U87MG malignant glioma cells were stably transfected to express luciferase using RediFect Red-FLuc lentiviral particles (Perkin Elmer). After anesthesia was established with 2% isoflurane, the mice were positioned in a stereotactic instrument. A burr hole was drilled into the skull 0.5 mm anterior and 2 mm lateral to the bregma using a 27-gauge needle. DAOY-Luc2 or U87MG-Luc2 cells (2 × 10^5^ cells in 5 μL PBS) were injected in the striatum and the mice were observed for stable tumor growth over two weeks as described previously [34]. The animals were imaged non-invasively using in vivo bioluminescence measurements to quantitate tumor growth. The tumor-bearing mice were injected with D-luciferin and bioluminescence was measured using an IVIS-200 Caliper Imaging System. The results were expressed as a total radiance in photons per sec/cm^2^ per steradian. Animals with stable and equivalent tumors in the brain were then divided randomly into two groups, the vehicle controls and those receiving SP-141 (dissolved in PEG400/ethanol/saline (57.1:14.3:28.6, *v*/*v*/*v*), 40 mg/day, 5 days a week). Tumor growth and body weights were monitored every 3 days. At the end of experiments, orthotopic tumors were excised, weighed, and snap-frozen for immunohistochemical staining.

### 2.7. Immunohistochemistry and Immunofluorescence Assays

Routine tissue fixation, paraffin embedding, and cutting into 5 μm sections was performed on tumor tissue specimens. For antibody staining, the tumor sections were blocked and incubated with specific antibodies (diluted 1:50 in 5% BSA in PBS) for 2 h at 23 °C. The slides were next incubated with streptavidin-peroxidase HRP conjugate and stained with DAB chromogen according to the manufacturer’s instructions (DACO Animal Research Kit). The final step involved light counterstaining. For detection of MDM2, p53, and p21cip1 by immunofluorescence, cells grown on glass coverslips were incubated with SP-141 for 12 h, fixed in 4% formaldehyde, and blocked with 5% goat serum and 0.3% Triton X-100 in PBS for 1 h. Next, the cells were incubated with primary antibodies, washed, and treated with FITC-conjugated secondary antibodies for 1 h. The images were photographed and quantitated using a multiphoton fluorescence microscope [30,31,32,33,34].

### 2.8. Statistics

All experiments were independently repeated at least three times, and the means of results or representative blots are presented. Two-sided t-tests were used for comparison between two groups. A value of *p* < 0.05 was considered statistically significant at a 95% confidence interval. A power analysis was used to calculate the minimum number of animals required to detect differences between tumor volumes assuming an 80% power, the desired P-value of 0.05, and a common standard deviation of 2.0. One-way ANOVA with Dunnett’s multiple comparisons test was performed for in vivo tumor efficacy studies.

## 3. Results and Discussion

Thus far, there have only been a few attempts to target p53 for glioma therapy, and these have been limited to the transfer of adenoviral wild-type *p53* gene constructs and replication-deficient oncolytic viruses to induce cell death [35,36]. However, there is a growing interest in targeting the MDM2 oncogene for treatment of human gliomas due to its frequent overexpression, the presence of p53 mutations, and abundant alterations of the p16INK4a/p15INK4b/ARF/MDM2 pathways that perturb the regulation of the tumor suppressor [8,9,10]. This is evident by the fact that several MDM2 inhibitors have recently been tested in this cancer type, including AMG-232, which has entered clinical trials [26,27,28]. However, it should be noted most of the MDM2 inhibitors developed to date, including AMG-232, have been designed to block the binding of MDM2 and p53, and are expected to have limited efficacy against tumors with mutant or deficient p53. Therefore, the studies reported here with SP-141 bear great significance, because of the drug’s ability to eliminate MDM2 irrespective of the p53 gene status.

### 3.1. SP-141 Shows Great Therapeutic Potential Against p53 wt and p53 Mutant Brain Tumor Cell Lines

**Figure 1:**
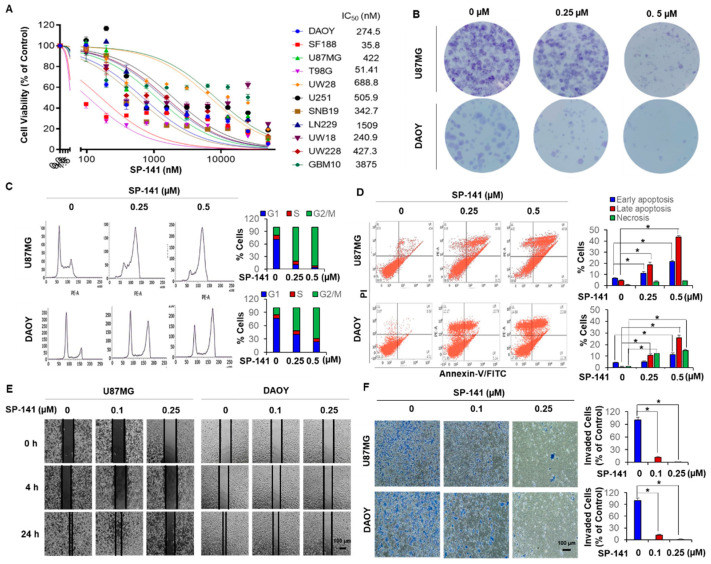
SP-141 shows therapeutic potential in p53 wt and p53 mutant brain tumor cell lines. (**A**) Cytotoxicity of SP-141 against brain tumor cell lines. Cell growth inhibition was analyzed by the resazurin reduction assay (data represent the results of three independent experiments performed in triplicate). Cells were treated with different dilutions of the compound (0–25 µM) for 72 h. Cytotoxicity (IC_50_) values in nM are stated for each cell line. (**B**) Colony formation of two representative cell lines. Cultured DAOY and U87MG cells were treated with SP-141 at different concentrations for 24 h. Twenty-one days after drug removal, cells were fixed and stained with crystal violet and images were prepared. (**C**) Analysis of cell cycle progression after treatment with different concentrations of SP-141 for 24 h. (**D**) Apoptosis analysis by flow cytometry using the Annexin V-FITC/PI stain assay. U87MG and DAOY cells were incubated with various concentrations of SP-141 for 24 h then were stained with membrane phosphatidylserine-binding Annexin V-FITC and propidium iodide (PI) (panels Lower Left: FITC Annexin V- and PI- negative; Lower Right: Annexin V-FITC positive; Upper Right: FITC Annexin V- and PI-positive; Upper Left: PI-positive) (* *p* < 0.01). (**E**) Cell migration. U87MG and DAOY cells were grown to confluence in six-well plates and a scratch was made at the start of the experiment. The cells were then exposed to SP-141 at the concentrations shown. The wells were imaged at different time points. Scale bars: 100 µm. (**F**) U87MG and DAOY cells were subjected to a Transwell invasion assay 12 h after treatment with SP-141 (0, 0.1, and 0.25 µM) as specified. The crystal violet dye staining images of lower chambers with migrated cells are shown (* *p* < 0.01). Scale bars: 100 µm.

SP-141 was rigorously assayed for its cytotoxic effects in a panel of nine GBM (SF188, U87MG, T98G, UW28, U251, SNB19, LN229, UW18, GBM10) and two medulloblastoma (DAOY and UW228) cell lines. The U87MG, LN229, GBM10, and UW28 cells harbor wild-type p53, while all of the others are known to contain p53 mutations, as shown in Supplementary Table S1. As shown in Figure 1A, all brain tumor cell lines tested were sensitive to the MDM2 inhibitor, with a wide range of cytotoxicity. While the pediatric GBM SF188 cells had a very low IC_50_ value of 35.8 nM, several GBM cell lines were in the mid-range of 250–500 nM and some GBM cells, such as the GBM10, were resistant with an IC_50_ of 3.875 µM. Significantly, we noted that SP-141 exerted similar levels of cytotoxicity towards the GBM and medulloblastoma cells, regardless of their p53 status, as shown in Figure 1A. The cell killing was confirmed by colony formation assays in U87MG and DAOY cells, which were chosen as representative p53 wild-type and mutant cell lines in this study, as shown in Figure 1B.

Since cell cycle progression is heavily influenced through p53 and p53-related signaling, the patterns of changes in the cell cycle were explored in DAOY and U87MG cells by propidium iodide staining. Representative histograms are shown in Figure 1C and reveal that there was a SP-141 concentration-dependent G2/M cell cycle phase block after 24 h (*p* < 0.01). Next, the cell death events occurring due to SP-141 exposure were quantitated by Annexin-V/propidium iodide staining under the same conditions used in the cell cycle experiments. The results, as shown in Figure 1D, showed a progressive concentration-dependent increase in number of Annexin-V-positive DAOY and U87MG cells, indicating the induction of early apoptotic events. There was a strong accumulation of dead cells (25.89% DAOY and 43.56% U87MG, *p* < 0.01) after 24 h of exposure to 0.5 µM SP-141, suggesting rapid induction of apoptosis due to MDM2 inhibition, irrespective of the p53 status of the cells. Together, the findings in Figure 1 indicate that cells arrested irreversibly at the G2/M phases by SP-141 may be channeled towards cell death. In addition, SP-141 also inhibited the migration, as shown in Figure 1E, and invasion, as shown in Figure 1F (*p* < 0.01), of these cells, confirming the critical role of MDM2 in anti-glioma activity.

### 3.2. SP-141 Downregulate MDM2 in p53 wt and p53 Mutant Brain Tumor Cell Lines

**Figure 2:**
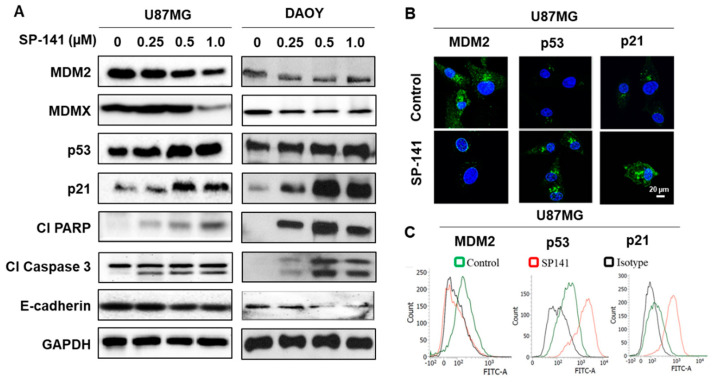
SP-141 downregulates MDM2 in p53 wt and p53 mutant brain tumor cell lines. (**A**) Brain tumor cells were treated with various concentrations of SP-141 for 24 h, then immunoblotting was performed to detect the levels of MDM2 and related proteins. GAPDH served as a loading control. (**B**) Immunofluorescence staining showing the expression of p53, MDM2, and p21 in SP-141-treated (0.5 µM for 12h) and untreated cells analyzed using specific p53, MDM2, and p21 antibodies. Scale bars: 20 µm. (**C**) Flow cytometric analysis of SP-141-treated (0.5 µM for 12h) U87MG cells using specific MDM2, p53, and p21 antibodies along with their isotype antibodies (IgG_1_, IgG_2a,_ and IgG, respectively) as isotype controls.

Our previous studies showed that SP-141 triggers a potent and specific elimination of MDM2 protein in breast cancer, pancreatic cancer, and hepatocellular carcinoma cells and the corresponding xenografts in a p53-independent manner [30,31,32]. To confirm these observations and to explore the possible clinical utility of SP-141 in brain tumors, we first determined the effect of SP-141 on MDM2, its targets, and apoptotic regulatory proteins. The p53 wt human glioblastoma U87MG and p53 mutant medulloblastoma DAOY cell lines were exposed to SP-141 (0.25–1 µM range) for 24 h, then Western blotting was performed to assess protein expression. Figure 2A shows a robust and SP-141 concentration-dependent upregulation of p21cip1, which was accompanied by a significant increase in the activated PARP and caspase 3 levels in both DAOY and U87MG cells. The increase in the p21 levels in response to SP-141 exposure is consistent with our previous findings that MDM2 is a p53-independent negative regulator of the CDK inhibitor [37]. The p53 protein showed a significant increase, while a distinct loss of the MDM2 was evident in both cell lines. The levels of E-cadherin, an epithelial-mesenchymal transition (EMT) regulator, and MDMX, an MDM2 partner which also negatively regulates p53, were also slightly reduced in both cell lines following SP-141 treatment, as shown in Figure 2A. Immunofluorescence studies confirmed that there was a detectable increase in the p53 and p21 levels, along with a loss of MDM2, in the U87MG cells, as shown in Figure 2B. Additionally, we performed a flow cytometric quantitation of these proteins using specific monoclonal antibodies and isotype IgG controls in U87MG cells treated with SP-141. There were clear peak shifts to the right for p53 and p21 and a leftward shift for MDM2 observed in these experiments, as shown in Figure 2C, confirming the increased p53 and p21 levels and elimination of MDM2 after SP-141 exposure.

### 3.3. SP-141 Shows Excellent Antitumor Activity in Orthotopic Glioblastoma and Medulloblastoma Xenograft Models

**Figure 3:**
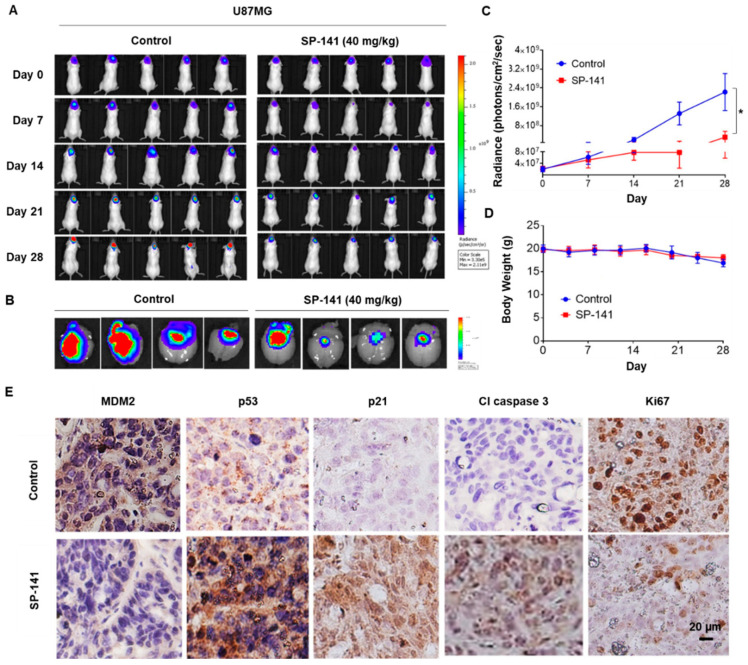
SP-141 shows excellent antitumor activity against U87MG human glioblastoma intracranial xenografts. (**A**) Representative images of bioluminescence acquisition of the brain tumors in nude mice after 0, 7, 14, 21, and 28 days of treatment. SP-141 was administered intraperitoneally at 40 mg/kg/day, 5 days per week for 4 weeks. SP-141 was dissolved in PEG400/EtOH/saline (57.1:14.3:28.6, *v*/*v*/*v*). The control group received the vehicle alone. (**B**) Ex-vivo bioluminescence from the brains of control (left panel) and SP-141-treated (right panel) mice. The scale indicates the fluorescence intensity in terms of radiant efficiency (p/sec/cm^2^/Sr). (**C**) Average time-dependent changes in bioluminescence radiance in U87MG xenografts in mice receiving SP-141 therapy over 4 weeks (* *p* < 0.01). (**D**) The average body weights of mice were plotted during SP-141 treatment. (**E**) Reduced MDM2 expression and activation of p53 signaling components were observed in U87MG intracranial xenografts following SP-141 treatment. Brain areas bearing the U87MG tumors from control and SP-141 treated nude mice were sectioned and processed for the expression of MDM2, p53, p21, cleaved caspase 3, and Ki67 proteins by immunohistochemistry. Representative staining patterns are shown. Scale bars: 20 µm.

Based on the powerful anticancer effects observed in vitro, we next assessed the in vivo efficacy of SP-141 in two orthotopic brain tumor models. Intracranial tumors were established by stereotaxic injection of Gaussia luciferase-expressing U87MG or DAOY cells in mouse brains, and the tumor growth was assessed by quantitative bioluminescence. SP-141 was administrated at 40 mg/kg/day, 5 days/week for 28 days to nude mice bearing an equivalent tumor burden. This dose of SP-141 was determined based on a previous pharmacokinetic study [29] and the efficacy studies performed in models of other cancer types [30,31,32]. The pharmacokinetic study indicated that SP-141 can cross the blood–brain barrier directly, with concentrations of the compound in the brain being higher than the in vitro IC_50_ values [29]. Representative color luminescent images reflecting the spatial distribution of photon counts of the U87MG glioblastoma cells in the brains of control and SP-141-treated nude mice are shown in Figure 3A. On days 21 and 28, there was a discernible decrease in the bioluminescence of treated mice, as shown in Figure 3A. This decrease (45 ± 2%) was particularly evident when the ex-vivo bioluminescence of brains from the SP-141-treated animals, as shown in the right panel of Figure 3B, was compared to that of controls, as shown in the left panel of Figure 3B. The quantitative changes in the average bioluminescence radiance calculated from eight animals bearing the U87MG tumors over the treatment period are indicated in Figure 3C. The findings indicate that there was a 4.5-fold decrease in tumor burden on day 28 compared to the control mice, as shown in Figure 3C (*p* < 0.01). There were no significant changes in the average body weight of tumor-bearing mice, as shown in Figure 3D, suggesting that SP-141 did not exert undue adverse effects on the host. Further, to verify the mechanism of action of SP-141 in vivo, we performed immunohistochemical staining of brain sections from control and drug-treated mice. As shown in Figure 3E, we observed a significant reduction of MDM2, a marked upregulation of p53, p21cip1, and cleaved caspase, and decreased levels of the proliferation marker Ki67. These results agree with the in vitro effects of SP-141 on U87MG cells, as shown in Figure 2A, and confirm its activity as an MDM2 inhibitor.

**Figure 4:**
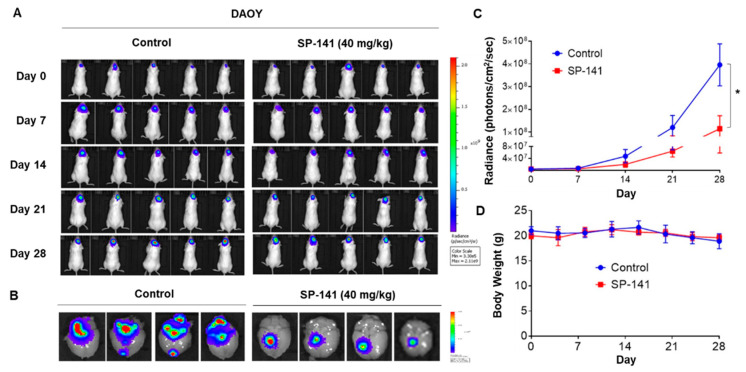
SP-141 shows excellent antitumor activity against DAOY human medulloblastoma intracranial xenografts. (**A**) Representative images of bioluminescence acquisition of brain tumors on day 0, 7, 14, 21, and 28 of treatment. The dose of SP-141 and treatment were the same as described in the legend to Figure 3A. (**B**) Ex-vivo bioluminescence from the brains of control (left row) and SP-141-treated (right row) mice. The scale indicates fluorescence intensity in terms of radiant efficiency (p/sec/cm^2^/Sr). (**C**) The average time-dependent changes in bioluminescence radiance in DAOY xenografts in mice receiving SP-141, treated over 4 weeks (* *p* < 0.01). (**D**) The average body weights of the mice were plotted during SP-141 treatment.

In addition, mice with the orthotopic DAOY medulloblastoma xenografts were treated with the same SP-141 dosage and schedule described above. The results from DAOY tumors are shown in Figure 4. The representative spatial distribution of photons in the animal brains is shown in Figure 4A, which revealed a significantly lower signal in the SP-141-treated group. Quantitation of the ex-vivo bioluminescence in mouse brains on day 28, as shown in Figure 4B, showed a 10.5-fold signal reduction in the SP-141-treated group. The average bioluminescence radiance changes from all eight animals in the group were determined and plotted against the treatment time and are shown in Figure 4C (*p* < 0.01). There was a 9-fold reduction in the tumor burden after SP-141. Again, there were no changes in the animal body weights during the treatment, reflecting the non-toxic nature of the drug. Of note, the DAOY xenografts were more sensitive than the U87MG glioblastoma, which was consistent with the IC_50_ of SP-141 against these cell lines, as shown in Figure 1A.

### 3.4. SP-141 Sensitizes Brain Tumor Cells to Temozolomide Treatment

**Figure 5:**
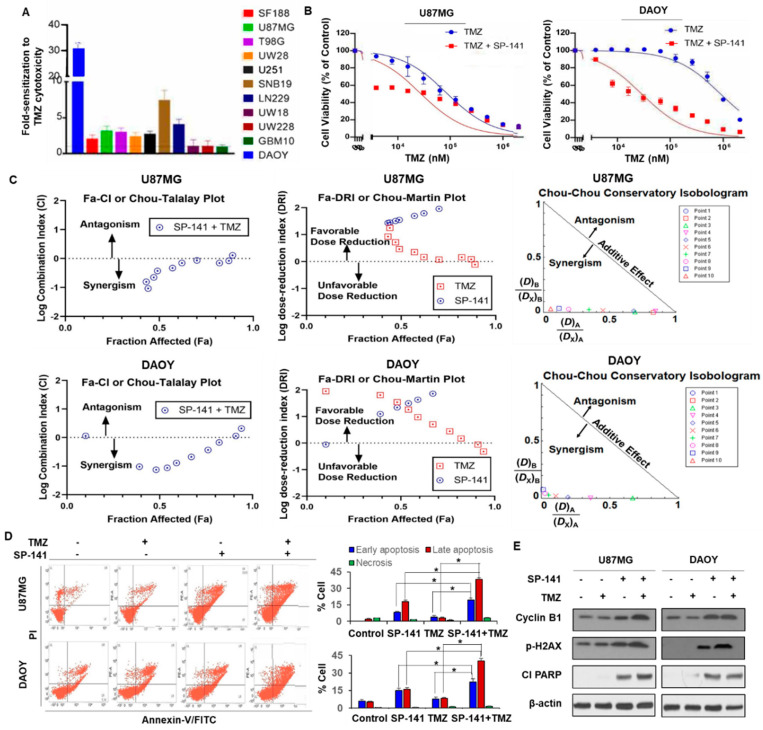
SP-141 sensitizes brain tumor cells to temozolomide treatment. (**A**) Bar graph showing the potentiation of TMZ cytotoxicity by SP-141 in a panel of 11 glioblastoma and medulloblastoma cell lines. The tumor cells were simultaneously exposed to SP-141 (at their respective IC_20_ values) and varying concentrations of TMZ (3 µM to 2 mM). The mean IC_50_ values of TMZ alone and those of the TMZ + SP-141 combinations were calculated in four independent cytotoxicity assays, each run in triplicate. The average fold-increase in cell killing (± SEM) for the TMZ + SP-141 combination was calculated and is represented in the bar graphs. (**B**) Potentiation of TMZ cytotoxicity by SP-141. The cytotoxicity of TMZ alone and in combination with SP-141 (at its IC_20_ concentration) against U87MG and DAOY brain tumor cells is shown. (**C**) Analyses of synergism in the cytotoxicity of the SP-141 + TMZ combination in DAOY and U87MG cells as determined by the CompuSyn software. The combination index (CI, Chou–Talalay plots) and dose-reduction index (DRI, Chou–Martin Plots) were generated according to the levels of growth inhibition (fraction affected, FA) by each drug used individually and in combination. The normalized isobologram showed that all 10 combination data points were located in the lower left, indicating synergism. The combination index (CI) = (D)A/(Dx)A + (D)B/(Dx)B where (D)A and (D)B were the concentrations of each drug alone required to exert x% effect, while (Dx)A and (Dx)B were the concentrations of drugs in combination to elicit the same effect. (**D**) Representative Annexin-V-FITC staining of DAOY and U87MG cells treated with 0.25 µM SP-141 and 60 µM TMZ, alone and in combination for 24 h. The mean percentage of cells in early and late apoptotic stages from four experiments of Annexin-V staining are shown in the bar graph. The differences in the early and late apoptotic populations in the cells treated with TMZ + SP-141 compared with TMZ alone or SP-141 alone were statistically significant (* *p* < 0.01). (**E**) The cells were treated with SP-141 and TMZ for 24 h prior to analyses of the protein expression.

TMZ is a small lipophilic alkylating agent that penetrates the blood–brain barrier and is the mainstay first-line chemotherapy drug used for GBM. Methylation at the O6-position of guanine is a key cytotoxic lesion generated by TMZ that serves to mutagenize the tumor genome and shrink the gliomas. However, brain tumors overexpress a DNA repair protein called MGMT (O6-methylguanine DNA methyltransferase), which repairs this lesion and prevents the mispairing of O6-methylguanine with thymine, and thereby effectively stops the cell killing mediated by TMZ [7]. In the absence of MGMT, the deficiency of mismatch repair is another determinant of glioma resistance to TMZ. Although powerful inhibitors of MGMT, such as O6-benzylguanine, can restore sensitivity to alkylating agents, this strategy has not been successful in the clinic because of the toxicity of DNA alkylation to the hematopoietic stem cells [38]. There is, therefore, an urgent need to develop alternative approaches to overcome the resistance of glioma to alkylating agents and/or to design new and effective drugs for this tumor type. Because MDM2 inhibitors and alkylating drugs act through different mechanisms, we examined whether there might be synergy between them. For this purpose, we exposed all of the brain tumor cell lines to TMZ over a large range with or without a sublethal SP-141 concentration (at the corresponding IC_20_ values) and determined the cell survival. The results showed a variable but significant increase in TMZ cytotoxicity ranging from a small 1.1-fold increase (GBM10, UW228, UW28) to a moderate 2- to 7-fold (SF188, U87MG, T98G, UW28, U251, LN229, SNB19) and to a high level of 31-fold potentiation in DAOY cells, as shown in Figure 5A. A comparative representation of TMZ sensitization by SP-141 in U87MG and DAOY cells is shown in Figure 5B. Although the increased cell killing seen in the presence of SP-141 + TMZ indicates possible synergy, the drug interactions require further validation because of the clinical importance of the findings. An isobologram analysis is an established method used to determine whether two drugs act additively, synergistically, or antagonistically [39]; this analysis is independent of the shape of the dose-response curves and allows for proper and quantitative classification of the degree of interaction between drugs. Therefore, we used the recent version of CompuSyn software (www.combosyn.com) developed based on Chou and Talalay’s findings [39]. Briefly, the combination index (CI) values were calculated according to the growth inhibition levels (fraction affected) by induced SP-141 or TMZ individually and in combination (SP-141 at its IC_20_ + varying TMZ concentrations). In this method, CI values < 1, = 1, or >1 would mean synergistic, additive, or antagonistic activity, respectively. The Chou–Talalay plots for both DAOY and U87MG cells, as shown in Figure 5C, showed that most CI values were < 1, indicating drug synergism. Rearranging the equation also provided algorithms to simulate the dose-reduction index (DRI) as shown in the Chou–Martin plots, as shown in Figure 5C. A strong dose-reduction of TMZ (DRI >1) was highly favored, suggesting a means to decrease the toxicity associated with this alkylating agent. The normalized (conservatory) isobolograms (Chou–Chou plots) drawn from 10 combination data points also allowed visualization of the drug synergism in both cell lines, as shown in Figure 5C. Collectively, the CompuSyn analyses confirmed that there was robust synergism between the SP-141 and TMZ in vitro. In addition, it is important to note that both the sensitivity to SP-141 as a single agent, as shown in Figure 1A, and sensitization to TMZ, as shown in Figure 5A,B, occurred in either the presence or absence of MGMT (Supplementary Table S1), indicating that the effects of SP-141 do not involve changes in DNA repair and are p53-independent.

To further explore the mechanism underlying this increased sensitization, we performed Annexin-V staining under the same conditions. U87MG and DAOY cells were exposed to 0.25 µM SP-141 and 60 µM TMZ (which approximate sub-lethal drug concentrations for both drugs alone) for 20 h before determining the extent of apoptosis. The results showed that the combination increased the rate and extent of cell death in both cell lines compared to either drug alone, as shown in Figure 5D. The percentage of cells that were in early or late apoptosis in four independent experiments showed that both parameters were significantly increased, reflecting a synergistic drug action (*p* < 0.01), as shown in Figure 5D. Western blot analyses demonstrated that the levels of cyclin B1 (a marker of G2/M cell cycle arrest), phosphorylated histone H2AX (a DNA damage indicator), and cleaved PARP were slightly increased in the cells treated with SP-141 + TMZ, as shown in Figure 5E, again supporting apoptosis as one of the contributors to the upsurge in cell killing. These observations are significant and merit further examination in a xenograft setting to verify the increased antitumor efficacy. Further detailed analyses and investigations are also required to identify the mechanisms.

## 4. Conclusions

This work demonstrates that MDM2 inhibition by SP-141 can effectively curtail the growth of brain tumors in vitro and in vivo, regardless of the p53 status of the tumor. The design and application of new drugs to fight the CNS tumors is the need of the hour, particularly because the current therapies are restricted to hydrophobic alkylating agents, and are hindered by the expression of MGMT. In this context, SP-141 was able to sensitize gliomas to TMZ, and this occurred independently of the MGMT status. The increased cell killing induced by the SP-141 + TMZ combination was shown to occur through enhanced apoptosis. Our observations are consistent with a previous report that MDM2 inhibition by nutlin3a enhanced the efficacy of TMZ in glioblastomas [40]. Collectively, the results of this study support the further development of SP-141 and/or its derivatives as innovative MDM2-targeted therapeutic strategies for combating brain tumors.

## Supplementary Materials

The following are available online at https://www.mdpi.com/2073-4409/9/7/1592/s1, Table S1: *p53*-status and *MGMT* proficiency of the brain tumor cell lines used in the study.
