# Supplementary material for: Targeted Brain Tumor Therapy by Inhibiting the MDM2 Oncogene: In Vitro and In Vivo Antitumor Activity and Mechanism of Action
Source: Cells. 2020 Jul 1;9(7):1592. doi: 10.3390/cells9071592 (PMC7408354; doi:10.3390/cells9071592)
Supplement: Supplementary file 1 [file cells-09-01592-s001.pdf]

SUPPLEMENTARY DATA

Targeted Brain Tumor Therapy by Inhibiting the MDM2 Oncogene: In vitro and in vivo Antitumor Activity and Mechanism of Action

Table 1. p53-status and MGMT proficiency of the brain tumor cell lines used in the study

Cell line	Subtype	p53 status	Reference	MGMT Presence
SF188	GBM	Mutant, G266E	[1]	Yes
U87MG	GBM	Wild-type	[2]	No
T98G	GBM	Mutant M237I	[3]	Yes
UW28	GBM	Wild type	[4]	Yes
U251	GBM	Mutant, R273H	[5]	No
SNB19	GBM	Mutant, R273H	[6]	No
LN229	GBM	Wild-type	[3]	yes
UW18	GBM	Mutant (Unknown)	Pers. Commun, Dr. F. Ali-Osman	yes
UW228	MB	Mutant, T155N	[7]	yes
GBM10	GBM	Wild-type	Pers. Commun, Dr. Jann Sarkaria	yes
DAOY	MB	Mutant, C242F	[8]	yes

References:

[1] Chen, P., Iavarone, A., Fick, J., Edwards, M., Prados, M., Israel, M.A. Constitutional p53 mutations associated with brain tumors in young adults. *Cancer Genet Cytogenet*. **1995,** *82*,106-115.

[2] Forbes, S., Clements, J., Dawson, E., Bamford, S., Webb, T., Dogan, A., Flanagan, A., Teague, J., Wooster, R., Futreal, P.A., Stratton, M.R. COSMIC 2005. *Br J Cancer*. **2006,** *94*, 318-322.

[3] Van Meir, E.G., Kikuchi, T., Tada, M., Li, H., Diserens, A.C., Wojcik, B.E., Huang, H.J.S., Friedmann, T., Thbolet, N.D., Cavenee, W.K. Analysis of the p53 gene and Its expression in human glioblastoma cells. *Cancer Res.* **1994,** *54*, 649-652.

[4] Lo, H.W., Stephenson, L., Cao, X., Milas, M., Pollock, R., Ali-Osman, F. Identification and functional characterization of the human glutathione S-transferase P1 gene as a novel transcriptional target of the p53 tumor suppressor gene. *Mol Cancer Res.* **2008,** *6,* 843–850.

[5] http://p53.free.fr/Database/p53_database_distr.html

[6] Fujiwara, T., Mukhopadhyay, T., Cai, D.W., Morris, D.K., Roth, J.A., Grimm, E.A. Retroviral-mediated transduction of p53 gene increases TGF-beta expression in a human glioblastoma cell line. *Int J Cancer.* **1994**, *56,* 834-839.

[7] Kunkele, A., Preter, K.D., Heukamp, L., Pajtler, K.J., Hartmann, W., Mittelbronn, M., Grotzer, M.A., Deubzer, H.E., Speleman, F., Schramm, A., et al. Pharmacological activation of the p53 pathway by nutlin-3 exerts anti-tumoral effects in medulloblastomas. *Neuro-Oncology.* **2012,** *14,* 859–869.

[8]. Raffel, C., Thomas, G.A., Tishler, D.M., Lassoff, S., Allen, J.C. Absence of p53 mutations in childhood central nervous system primitive neuroectodermal tumors. *Neurosurgery*. **1993,** *33*,301–305.
